# *In situ* formed scaffold with royal jelly-derived extracellular vesicles for wound healing

**DOI:** 10.7150/thno.84665

**Published:** 2023-05-08

**Authors:** Dehong Tan, Wenxiang Zhu, Lujie Liu, Yuanwei Pan, Yangtao Xu, Qinqin Huang, Lingling Li, Lang Rao

**Affiliations:** 1 The Research and Application Center of Precision Medicine, The Second Affiliated Hospital of Zhengzhou University, Zhengzhou 450014, China.; 2 Institute of Biomedical Health Technology and Engineering, Shenzhen Bay Laboratory, Shenzhen 518132, China.; 3 Department of Pharmaceutics, School of Pharmacy, Nanjing Medical University, Nanjing 211166, China.; 4 College of Materials Science and Engineering, Hunan University, Changsha 410082, China.; 5 Cancer Center and Department of Oncology, Renmin Hospital of Wuhan University, Wuhan 430060, China.

**Keywords:** Hydrogel, Royal Jelly, Extracellular Vesicles, Wound Healing, Biomaterials

## Abstract

**Background:** Safe and effective wound healing can be a major clinical challenge. Inflammation and vascular impairment are two main causes of inadequate wound healing.

**Methods:** Here, we developed a versatile hydrogel wound dressing, comprising a straightforward physical mixture of royal jelly-derived extracellular vesicles (RJ-EVs) and methacrylic anhydride modified sericin (SerMA), to accelerate wound healing by inhibiting inflammation and promoting vascular reparation.

**Results:** The RJ-EVs showed satisfactory anti-inflammatory and antioxidant effects, and significantly promoted L929 cell proliferation and migration *in vitro*. Meanwhile, the photocrosslinked SerMA hydrogel with its porous interior structure and high fluidity made it a good candidate for wound dressing. The RJ-EVs can be gradually released from the SerMA hydrogel at the wound site, ensuring the restorative effect of RJ-EVs. In a full-thickness skin defect model, the SerMA/RJ-EVs hydrogel dressing accelerated wound healing with a healing rate of 96.8% by improving cell proliferation and angiogenesis. The RNA sequencing results further revealed that the SerMA/RJ-EVs hydrogel dressing was involved in inflammatory damage repair-related pathways including recombinational repair, epidermis development, and Wnt signaling.

**Conclusion:** This SerMA/RJ-EVs hydrogel dressing offers a simple, safe and robust strategy for modulating inflammation and vascular impairment for accelerated wound healing.

## Introduction

The skin is one of the largest, most exposed, and vulnerable organs in the human body, with multiple functions such as secretion, protection, and detection of tactile stimuli 1,2. However, the integrity of normal skin tissue structure and physiological function can be damaged by minor and major trauma, including surgery 3. In individuals with impaired wound healing, chronic wounds caused by inflammation and impaired vascularity have become a major medical challenge 4. Therefore, it is clinically important to develop wound dressings that address these two problems. Nanomaterials have recently become widely used in wound healing due to their excellent drug loading, adsorption, and antibacterial properties 5,6. A variety of stem cell exosomes have been used to effectively promote wound repair 7-9. However, the production of exosomes is costly and complicated, limiting their clinical applications.

Royal jelly (RJ) is a viscous slurry secreted by the developed hypopharyngeal head gland and maxillary gland of young worker bees 10,11. The RJ is an essential nutritional food for the growth, development and metabolism of queen bees and bee larvae 12-14, in humans it is often used in traditional medicine and natural health products due to its anti-bacterial, anti-inflammatory, anti-tumor and antioxidant effects 15. Extracellular vesicles (EVs), membrane-bound vesicles released by cells into the extracellular space, have crucial functions in cell-cell communication and control a variety of biological processes 16-20. Very recently, the EVs derived from RJ (RJ-EVs) have been reported to retain most of the effects of RJ and positively influence wound healing 21,22. In addition, the RJ-EVs have been reported to increase the migration of mesenchymal stem cells (MSCs) and fibroblasts 23,24. However, after local administration, it is difficult to retain RJ-EVs in the wound for a long time, which reduces the curative effect. Therefore, new delivery systems need to be developed for sustained release of RJ-EVs at the wound site.

Hydrogels can form a physical barrier at the bleeding site, help stop bleeding, maintain a moist wound environment for debriding, and allow oxygen penetration while maintaining good biocompatibility, making them effective wound dressings 25,26. Currently, various hydrogels prepared by synthetic or natural polymers, such as collagen and gelatin, have been used for skin repair 27,28. However, the mechanical stability of collagen-based hydrogels is poor, and gelatin and its derivatives are only soluble in hot water, limiting their use. Hydrophilic sericin extracted from silkworm cocoons has attracted much attention due to its biocompatibility, biodegradability, anti-tyrosinase, anti-coagulation, and anti-aging properties 29. More importantly, sericin has been demonstrated to stimulate fibroblast and keratinocyte growth and proliferation 30,31. Methacrylic anhydride (MA) modified sericin (SerMA) solutions have remarkable injectability, adhesion, and good biocompatibility, suggesting a potential use for SerMA in encouraging wound healing 30,31. In addition, MA modified sericin has the following advantages: (1) The MA-modified sericin hydrogel experimental method is simple and easy to scale up; (2) The MA is readily available, affordable, and has been produced commercially; (3) The MA-modified sericin to have a "C=C" double bond and thus light curing ability. In brief, the sol-gel transformation can be accomplished by irradiating the MA-modified sericin hydrogel prepolymer solution with a 405 nm flashlight.

**Figure 1:**
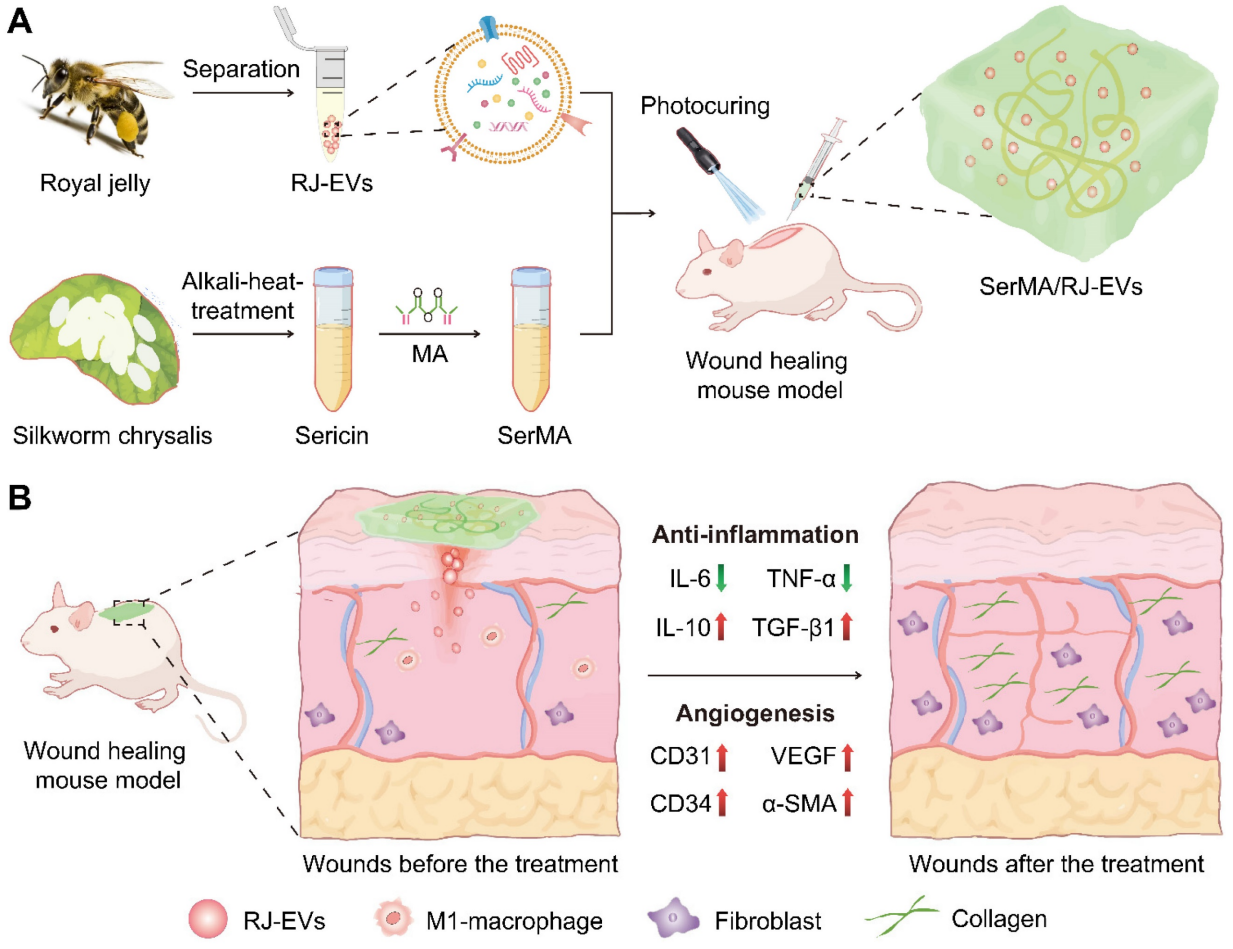
Schematic illustration of experimental procedure of the *in vivo* study. (A) The preparation of SerMA/RJ-EVs. (B) The SerMA/RJ-EVs hydrogel modulates inflammation and vascular impairment for accelerated wound healing.

In this study, we developed a SerMA/RJ-EVs hybrid hydrogel dressing, by simply mixing SerMA and RJ-EVs, to improve the wound repair (Figure 1A). The SerMA modified by MA has a high water content, low immunogenicity, and its structure is similar to the natural extracellular matrix (ECM), which may mean that it has a positive effect on the healing of damaged tissues. Meanwhile, the photocrosslinking property of the SerMA hydrogel enables spatiotemporal control of *in situ* gelation. In this study, the RJ-EVs in the SerMA hydrogel dressings showed excellent sustained release *in vivo*. More importantly, based on a full-thickness skin defect model, the SerMA/RJ-EVs hydrogel dressing significantly accelerated wound healing with a healing rate of 96.8% by encouraging cell proliferation and angiogenesis (Figure 1B). The RNA sequencing results revealed that the SerMA/RJ-EVs hydrogel dressing was involved in inflammatory damage repair-related pathways including recombinational repair, skin epidemis development, and Wnt signaling. The SerMA/RJ-EVs hydrogel dressing has great potential for use in clinical wound care, and prospectively, it may also be applied for treatment of other diseases.

## Results and Discussion

### Preparation and Characterization of RJ-EVs

**Figure 2:**
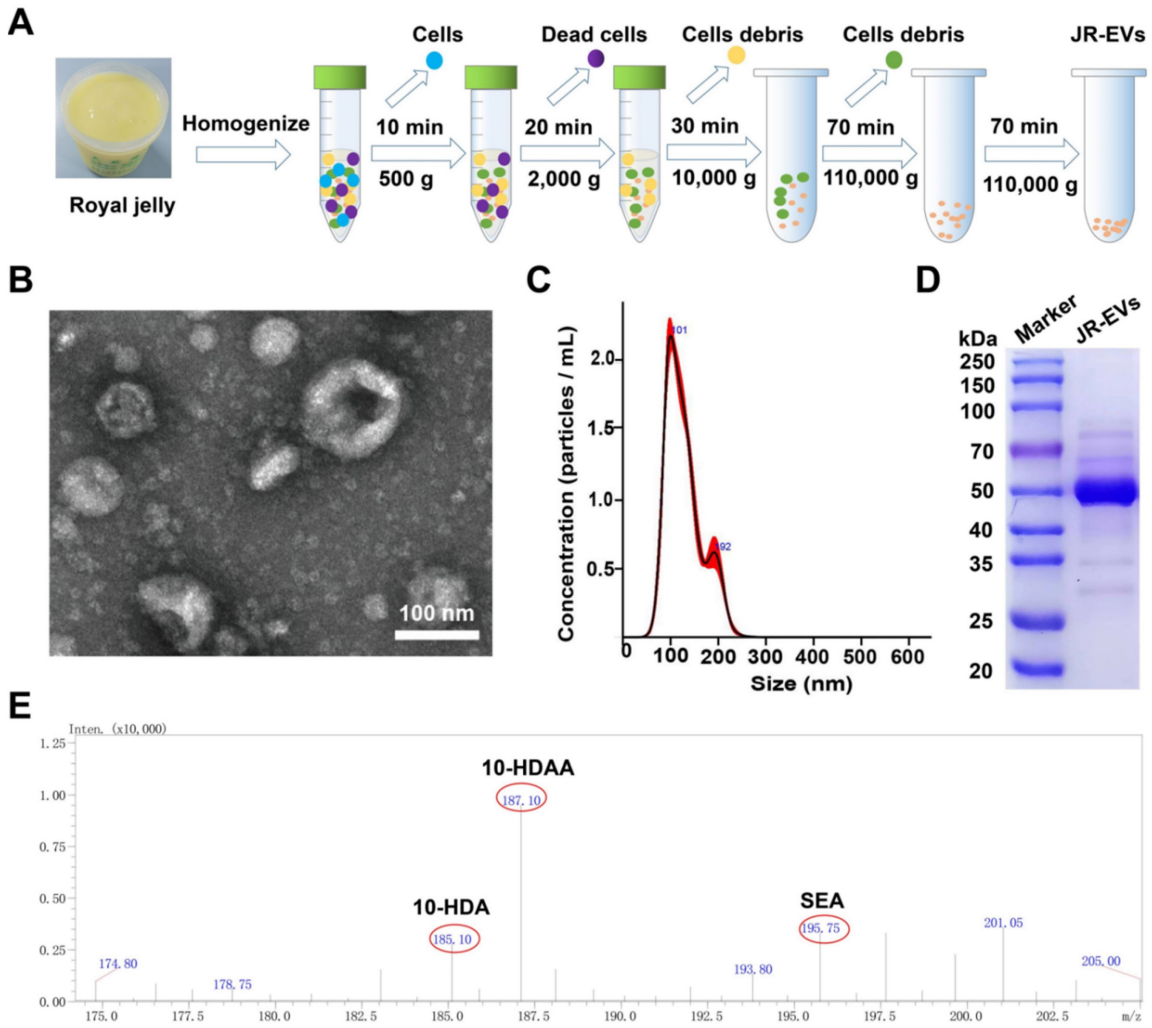
Isolation and characterization of RJ-EVs. (A) Schematic drawing of the gradient centrifugation protocol for isolating RJ-EVs from RJ. (B) TEM of RJ-EVs. (C) NTA analysis of RJ-EVs. (D) The SDS-PAGE of RJ-EVs proteins. Proteins were run on a 12% Bis-Tris protein gel and visualized with Coomassie blue. (E) The LC-MS spectrum analysis of free fatty acid species from RJ-EVs. Three representative fatty acids in RJ: 10-Hydroxy-2-decenoic acid (10-HDA), 10-Hydroxydecanoic acid (10-HDAA), sebacic acid (SEA).

The RJ-EVs were isolated from fresh royal jelly and purified by gradient ultracentrifugation. (Figure 2A). To observe the morphology of RJ-EVs, appropriate concentrations of RJ-EVs were negatively stained with a 1% uranyl acetate solution and captured by transmission electron microscopy (TEM) (Figure 2B). TEM showed a typical saucer-like bilayer structure, and the average size of RJ-EVs was determined to be about 100 nm in diameter, consistent with the result of the nanoparticle tracking analysis (NTA) (Figure 2C) and the dynamic light scattering (DLS) (Figure S1). Further, we investigated the stability of RJ-EVs and the results showed that there was no significant change in the hydrodynamic size of RJ-EVs in PBS for 9 days (Figure S2). The mean zeta potentials of RJ-EVs were about -15.12 mV (Figure S3) because both EVs and the plasma membrane of cells possess a negative surface charge when suspended in a neutral medium. The results of SDS-polyacrylamide gel electrophoresis (SDS-PAGE) further showed that the major bands ranged from 49 to 87 kDa (Figure 2D), which was consistent with the molecular weight range of major royal jelly proteins (MRJPs). MRJPs belong to a protein family with high homology, and 9 family members (MRJP1~9) have been verified at cDNA and protein levels 32. Among them, MRJP1~5 is the most abundant protein in RJ, accounting for 82%-90% of the total protein. SDS-PAGE showed that the expression level was highest around 49~55 kDa, suggesting that RJ-EVs are also rich in MRJP1 and MRJP2 10. In addition, MRJP3 (60-70 kDa) was detected, and many studies have confirmed that it can promote cell proliferation and wound healing 33,34. Royal jelly has a variety of biological functions not only related to its rich proteins but also its unique lipids. Lipids have been successfully identified in RJ-EVs by thin layer chromatography (TLC) (Figure S4) 21. Mass spectrometry (LC-MS) analysis showed that the RJ-EVs contained 10-hydroxy-decanoic acid (10-HDAA), sebacic acid (SEA) and trans-10-hydroxy-2-decanoic acid (10-HDA) (Figure 2E). Due to the special structure of these fatty acids, royal jelly fatty acids have a wide range of biological activities, such as anti-inflammation, neuroprotection, antioxidation, antibacterial protection, etc 35. In short, RJ-EVs contain favorable RJ-specific biologically active fatty acids and proteins, confirming their role in promoting wound healing.

### Pro-Proliferative and Antioxidant Properties of RJ-EVs

**Figure 3:**
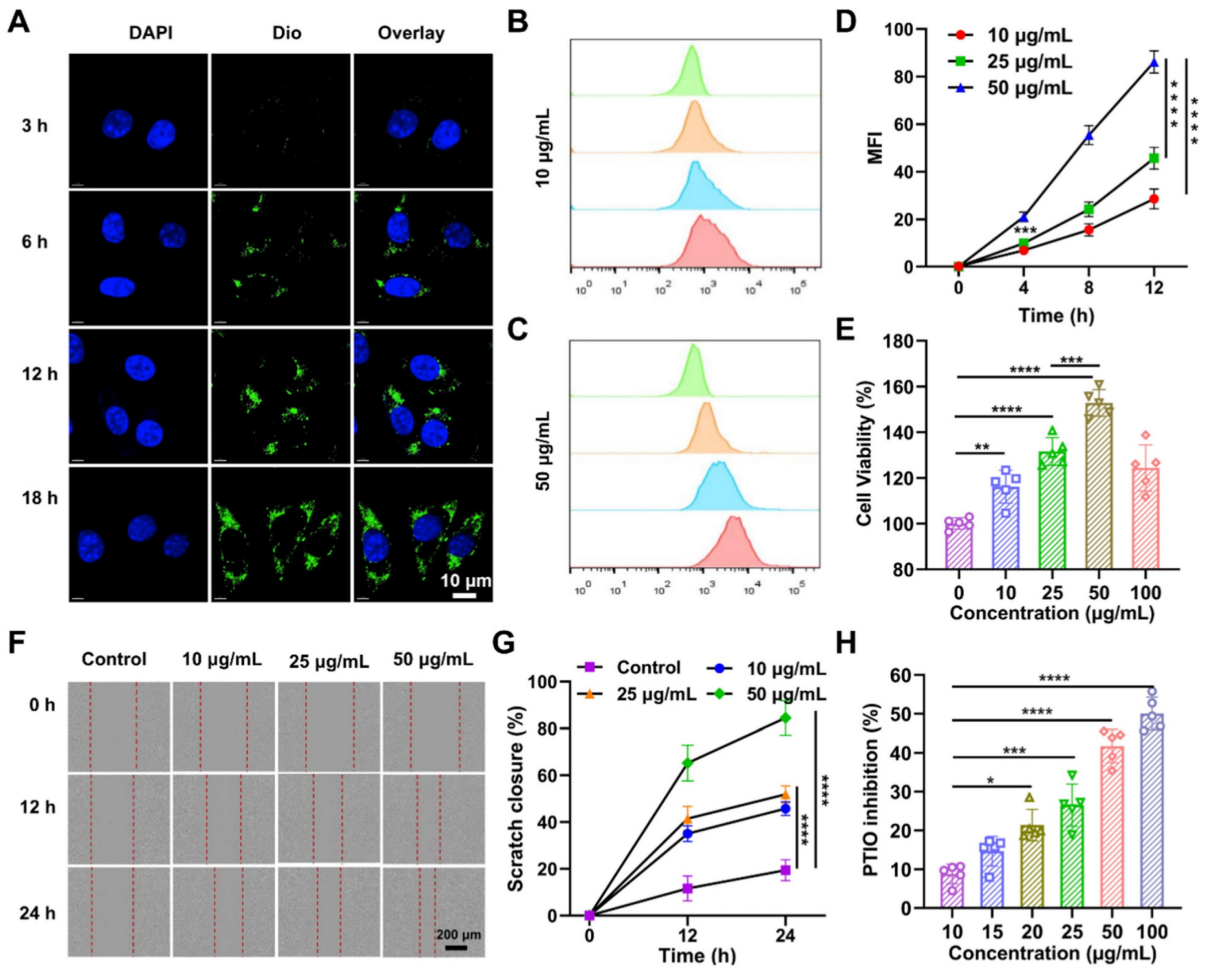
Interaction of RJ-EVs and mouse fibroblasts L929 cells *in vitro*. (A) CLSM of the intracellular uptake analysis RJ-EVs of mouse fibroblasts L929 cells under different treatments. Blue signals: DAPI-labeled nuclei; green signals: DiO-labeled RJ-EVs. Flow cytometry detection (B, C) and quantitative statistics (D) of the RJ-EVs in mouse fibroblasts L929 cells. (E) Viabilities of mouse fibroblasts L929 cells after incubation with RJ-EVs at different concentrations for 24 h. (F) Cell migration-promoting ability of RJ-EVs. (G) Quantitative statistics of the cellmigration rate. (H) Antioxidant property of RJ-EVs, PTIO assay to detect the scavenging ability against of DPPH radicals. All data were presented as mean ± SD (*n* = 5). * for *P* < 0.05, ** for *P* < 0.01, *** for *P* < 0.001, and **** for *P* < 0.0001.

Before investigating the effects of RJ-EVs on the cell proliferation of mouse fibroblasts (L929 cells), we observed the cellular uptake of RJ-EVs by L929 cells via confocal laser scanning microscopy (CLSM) 36. After incubation with 10 μg/mL RJ-EVs for 3 h, 6 h, 12 h, and 18 h, the cells exhibited strong green fluorescence under excitation at 480 nm (Figure 3A). These results suggested that RJ-EVs can enter cells in a time-dependent manner. In addition, flow cytometry results showed that the RJ-EVs entered cells in a concentration-dependent manner (Figure 3B-D, S5 and S6).

To investigate the effects of RJ-EVs on cell proliferation, the L929 cells were treated with various concentrations of RJ-EVs for 24 h. The results of CCK-8 assay showed that with an increasing concentration, the pro-proliferative effect of RJ-EVs was significantly enhanced, peaking at RJ-EVs concentration of 50 μg/mL (Figure 3E). Decreased cell viability was observed after treatment with 100 μg/mL RJ-EVs, likely resulting from limited cellular uptake efficiency. Furthermore, a cell scratch test revealed that RJ-EVs could significantly improve the migration ability of L929 cells while also effectively promoting the scratch healing (Figure 3F and 3G).

The PTIO (2-Phenyl-4,4,5,5-tetramethylimidazoline-3-oxo-1-oxo) method was used to evaluate the antioxidative efficiency of RJ-EVs 37-39 and the results showed that the antioxidant capacity of exosomes was concentration-dependent (Figure 3H). When the concentration of exosomes reached 100 μg/mL, the antioxidant efficiency was about 50%. Patients with wound infections frequently experience oxidative stress, exacerbated by insufficient antioxidant protection, which suppresses cell proliferation. The RJ-EVs' antioxidant activity thus likely contributes to cell proliferation and the overall healing process. The antioxidant effect of RJ may be related to the presence of free amino acids such as lysine, proline, cysteine, and aspartic acid. At present, the antioxidant mechanism of RJ-EVs is still unclear, and further studies are needed to discover the relevant antioxidant mechanism 38,39.

### Anti-Inflammatory Effects of RJ-EVs

**Figure 4:**
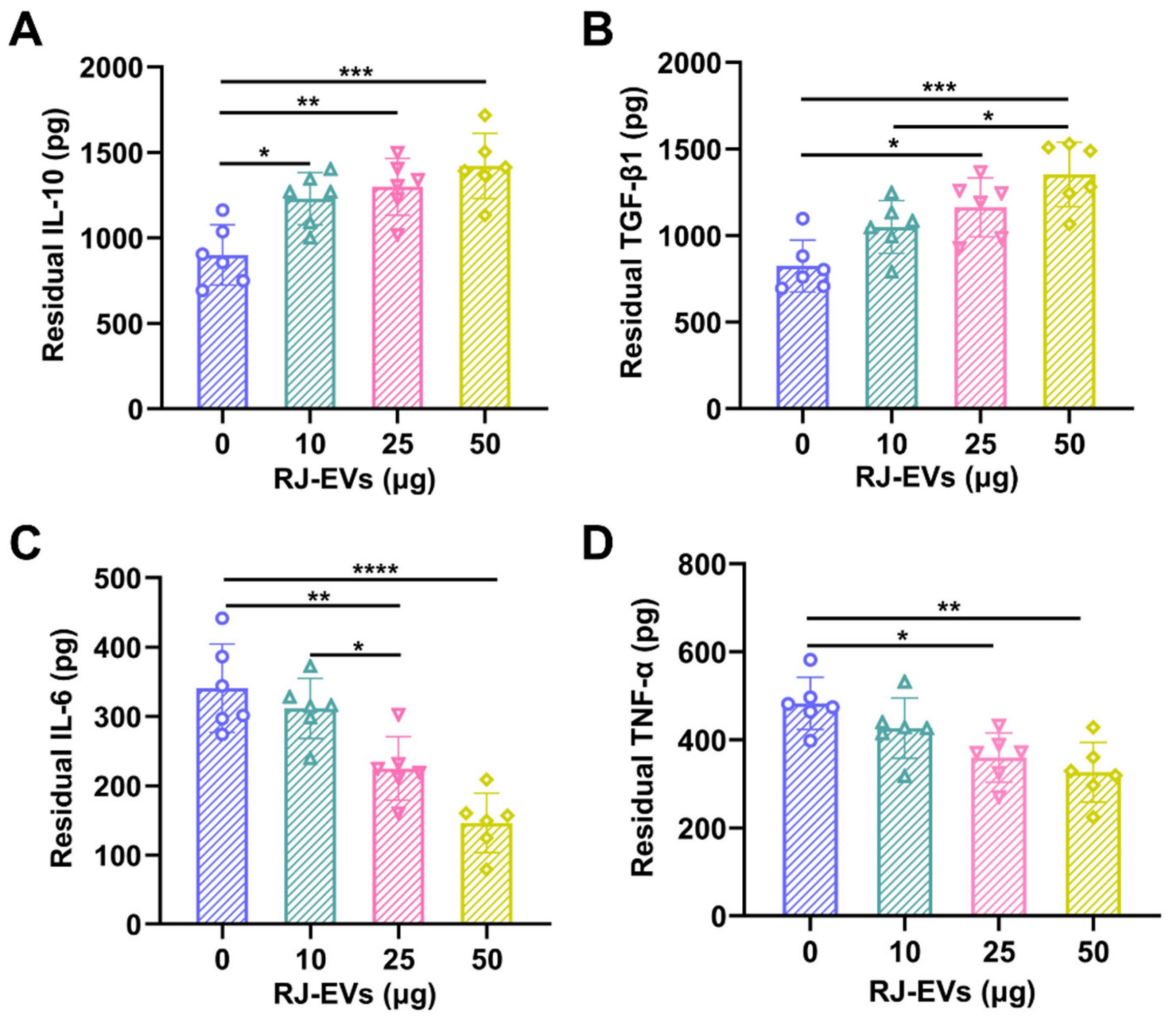
*In vitro* anti-inflammatory activities of RJ-EVs on Raw 264.7 macrophages. (A, B) The concentrations of anti-inflammatory cytokines (IL-10, TGF-β1) were quantified using ELISA assay. (C, D) The concentrations of pro-inflammatory cytokines (IL-6, TNF-α) were quantified using ELISA assay. All data were presented as mean ± SD (*n* = 6). * for *P* < 0.05, ** for *P* < 0.01, *** for *P* < 0.001, and **** for *P* < 0.0001.

Many typical pro-inflammatory cytokines, produced and secreted by macrophages, are in the long run negatively involved in the wound healing process. To evaluate the anti-inflammatory activity of RJ-EVs, an *in vitro* model of inflammation was established in RAW 264.7 cells stimulated with lipopolysaccharides and treated with different concentrations (*i.e.*, 0, 10, 25, 50 μg/mL) of RJ-EVs for 24 h. ELISA were performed to detect the common pro-inflammatory TNF-α and IL-6 and the anti-inflammatory IL-10 and TGF-β1 40. Compared with the control group, the expression levels of anti-inflammatory factors IL-10 and TGF-β1 in the experimental group were significantly increased (Figure 4A and 4B), and the expression levels of pro-inflammatory factors TNF-α and IL-6 decreased significantly in the experimental group (Figure 4C and 4D). The above results suggest that the RJ-EVs provide an effective method for the treatment of inflammatory diseases.

### Synthesis and Characterization of SerMA/RJ-EVs

**Figure 5:**
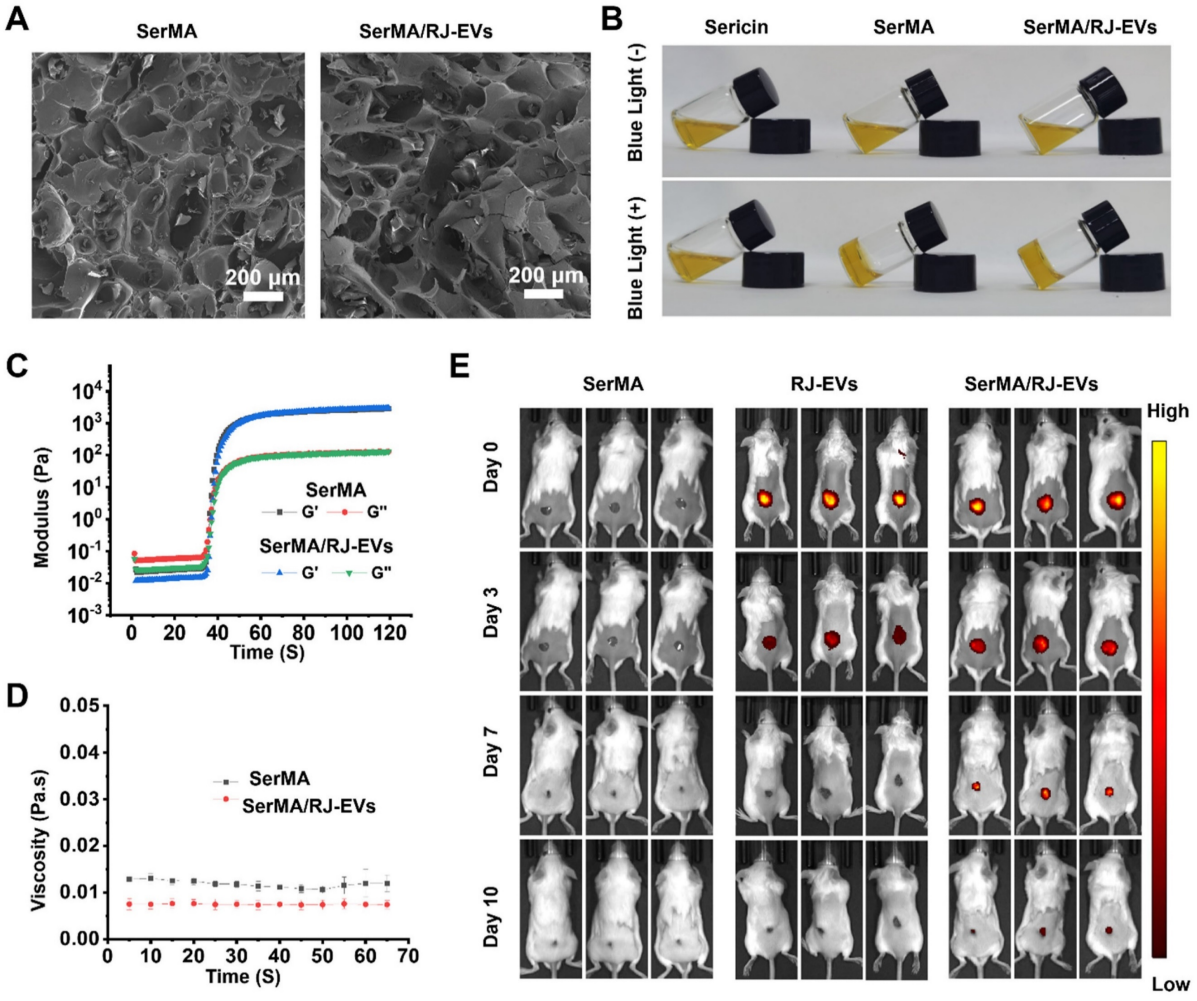
Fabrication and characterization of SerMA and SerMA/RJ-EVs. (A) SEM images of SerMA and SerMA/RJ-EVs lyophilized scaffold. (B) Photograph of the blue light-cured SerMA hydrogel. (C) Rheological properties of SerMA and SerMA/RJ-EVs. (D) The viscosity characterization of SerMA and SerMA/RJ-EVs. (E) *In vivo* distribution of DiR-labeled RJ-EVs in the SerMA/RJ-EVs hrdrogel dressings at different time point. All data were presented as mean ± SD (*n* = 5).

To investigate the effect of the incorporation of RJ-EVs on the structure of SerMA, the porous morphology of the hydrogel was observed by SEM (Figure 5A). The results showed that both SerMA and SerMA/RJ-EVs have obvious three-dimensional porous morphologies, indicating that the incorporation of RJ-EVs does not significantly affect the pore structure of the SerMA hydrogel 41. The precursor SerMA/RJ-EVs hydrogel solutions were prepared by mixing RJ-EVs (25 μg/mL), lithium phenyl-2,4,6-trimethylbenzoylphosphinate (LAP) (5 mg/mL), and SerMA (5 mg/mL) in phosphate-buffered saline (PBS). The SerMA and SerMA/RJ-EVs precursor hydrogel solutions were solidified by exposure to blue light at 405 nm, subsequently obtaining SerMA and SerMA/RJ-EVs hydrogels (Figure 5B). The 1H NMR spectra confirmed that MA is succesfully conjugated onto sericin as the new peaks occur at “C = C” (δ 5.7 ppm and 6.1 ppm) (Figure S7). The FTIR results showed that the characteristic peaks representing amide I (N-H and C-N bonds), amide II (N-H bonds) and amide III (C=O bonds) at 1255 cm^-1^, 1525 cm^-1^ and 1645 cm^-1^ were found in sericin and SerMA, indicating that the main structure of MA-modified sericin was not changed (Figure S8). The SerMA/RJ-EVs hydrogel had good adhesion to skin (Figure S9), which can effectively prevent the samples from falling off. In addition, the equilibrium swelling rates of SerMA and SerMA/RJ-EVs hydrogels were very similar, indicating that the doping of a small amount of RJ-EVs had little effect on the equilibrium swelling rate of SerMA hydrogels (Figure S10). Dynamic time-scan rheological analysis showed that SerMA and SerMA/RJ-EVs photocured within 40 seconds after exposure to 405 nm UV at a dose of 30 mW cm^-2^ (Figure 5C). In addition, the viscosity of SerMA and SerMA/RJ-EVs did not change much over time, indicating that the formed gel was stable (Figure 5D). The compressive stress-strain curves showed that the compressive strength of SerMA and SerMA/RJ-EVs was about 16 kPa (Figure S11). The apoptosis of L929 cells after SerMA/RJ-EVs hydrogel treatment was assessed in a transwell model (Figure S12). The experimental results showed that the SerMA/RJ-EVs hydrogel had no obvious pro-apoptotic effect on L929 cells, indicating that it had good biocompatibility. Furthermore, we assessed cell migration using SerMA hydrogels in a transwell model (Figure S13). The results showed that the SerMA hydrogel had no significant effect on cell migration. In addition, the SerMA/RJ-EVs hydrogels degraded slowly *in vitro* (Figure S14). The proper degradation rate of the hydrogel also showed its great application potential as a drug delivery system. We found no effect on the bioactivity of RJ-EVs irradiated by 405 nm visible blue light (Figure S15).

### Sustained Release of RJ-EVs from SerMA/RJ-EVs

Wound repair is a relatively slow process, and the long-term retention of RJ-EVs at the wound site is particularly important 42. Our *in vitro* studies showed that the RJ-EVs can be slowly released from SerMA/RJ-EVs (Figure S16). We could observe that the release efficiency of RJ-EVs was high in the first few days before leveling off; this explains why the SerMA hydrogel can be used as a wound dressing for long-term sustained RJ-EVs release 43. By labeling RJ-EVs with DiR dye (dark red fluorescence), we measured the degradation of the SerMA/RJ-EVs hydrogel and the sustained release of RJ-EVs *in vivo*. The SerMA hydrogel without RJ-EVs did not exhibit any red fluorescence, whereas the fluorescence of RJ-EVs persisted for 3 days and the fluorescence of the SerMA/RJ-EVs hydrogel persisted for 10 days (Figure 5E), indicating SerMA/RJ-EVs' ability to sustain wound healing. The therapeutic effect of the RJ-EVs alone were weaker than that of SerMA/RJ-EVs, because pure RJ-EVs form a non-adhesive droplet, which causes them to slide down from the wound surface, and only a small portion is taken up by cells. Quantitative analysis of relative fluorescence intensity at different time points was done and the SerMA hydrogel can thus slow the release of RJ-EVs for a long time and produce a sustained therapeutic effect on wounds (Figure S17).

### Skin Regenerative Activity of SerMA/RJ-EVs

**Figure 6:**
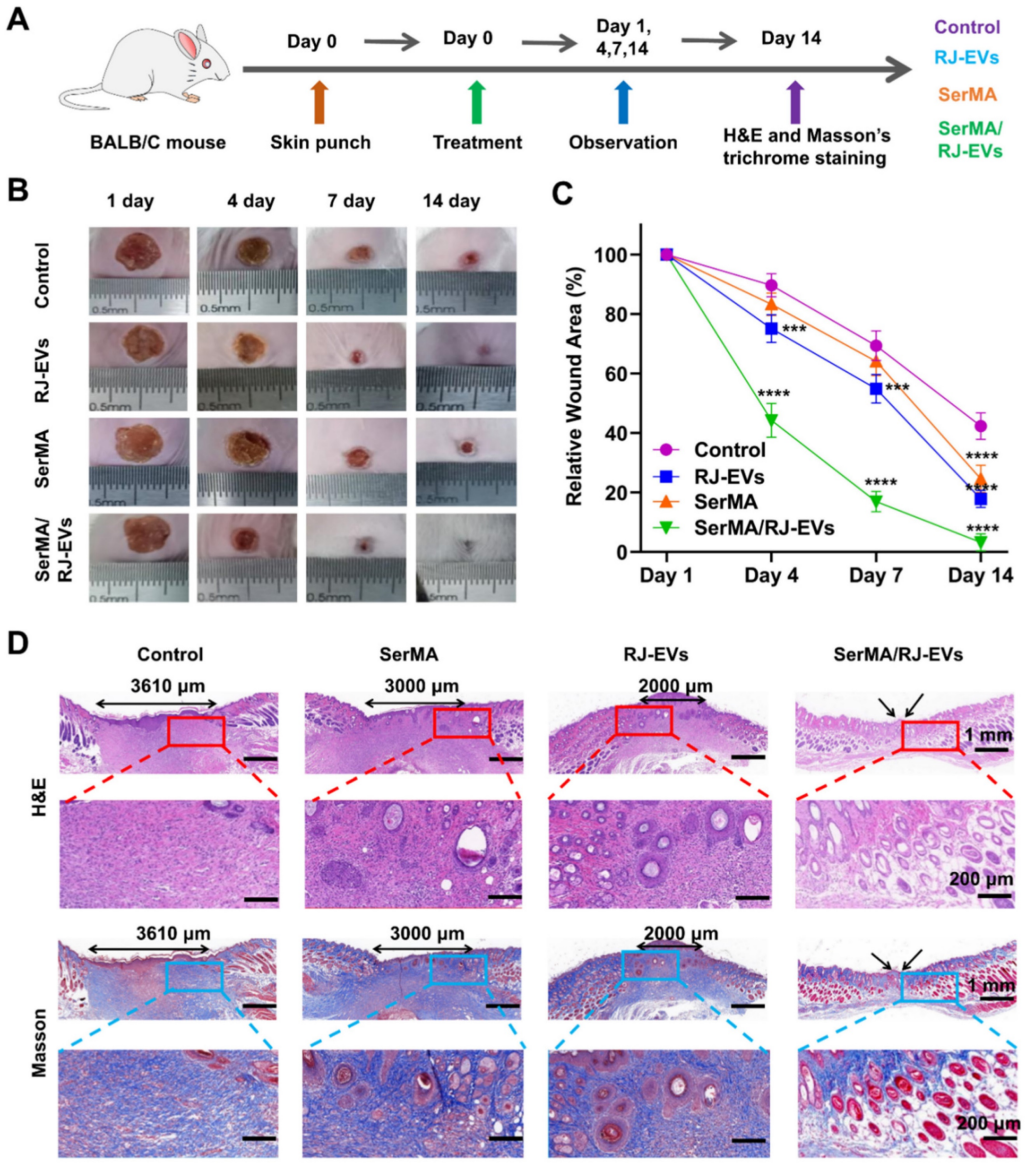
Healing process and histological evaluation of the SerMA/RJ-EVs in the mouse full-thickness wound model. (A) Schematically illustrated construction of the treatment procedure. (B) Representative images of the wound surface in four treated groups at day 1, 4, 7, and 14 post-treatment. (C) Relative wound area at different time points of four group. (D) Representative H&E and Masson staining images of different wound samples at day 14. Red and bule border image at the bottom of the panel is a partial enlargement of the image above. All data were presented as mean ± SD (*n* = 5). *** for *P* < 0.001, and **** for *P* < 0.0001.

To evaluate the promoting effect of SerMA/RJ-EVs hydrogel on wound healing* in vivo*, a full-thickness skin wound model was established in mice 44. A schematic diagram of full-thickness skin wound modeling and treatment is shown in Figure 6A. Representative photographs of wounds and traces of wound bed closure at days 0, 4, 7, and 14 are shown in Figure 6B. From these photos, it can be seen that the wound healing rate of the SerMA/RJ-EVs hydrogel was significantly higher than that of the control group, SerMA group and RJ-EVs group. The wound healing rates of the SerMA/RJ-EVs hydrogel group on the days 4 and 7 were 54.5% and 87.6%, respectively (Figure 6C); these rates were significantly higher than those of the control group, SerMA group and RJ-EVs group at the same time points. More importantly, after 14 days of treatment, the wounds in the SerMA/RJ-EVs hydrogel group were almost completely healed (96.8%), while the wound closure rates in the control and SerMA and RJ-EVs groups were 54.8%, 77.9%, and 80.6%, respectively. In addition, histological staining showed that the incorporation of RJ-EVs significantly promoted the formation of new skin tissue (Figure 6D). For the control group, the distance between newly regenerated skin tissue and mature tissue was approximately 3610 μm. The distances for the SerMA and RJ-EVs groups were reduced to 3000 μm and 2000 μm, respectively. The SerMA/RJ-EVs group thus exhibited the best wound healing efficiency with a negligible distance between newly formed tissue and mature tissue 45. Furthermore, Masson staining images showed more uniform collagen deposition in the SerMA/RJ-EVs hydrogel group than that in the other groups (Figure 6D). The above results indicated that the SerMA/RJ-EVs hydrogel dressing can significantly promote tissue regeneration and wound healing.

### Angiogenic Properties and Biosafety of SerMA/RJ-EVs

**Figure 7:**
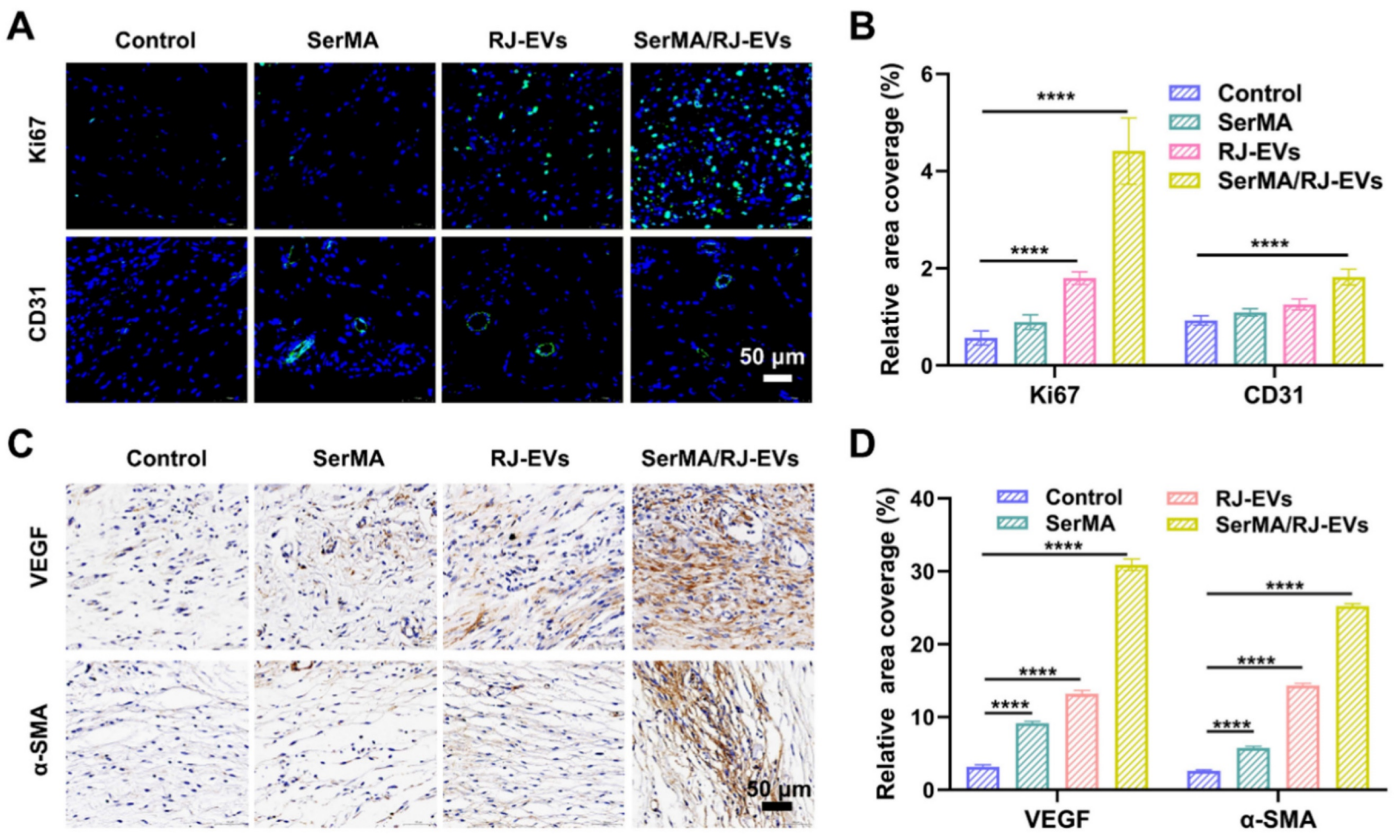
Healing mechanism of a full-thickness skin injury was analyzed by immunofluorescence staining and immunohistochemical staining. (A) Representative images of Ki67 (green fluorescence) and CD31 (green fluorescence) immunofluorescence staining at day 7 after different treatments. (B) Quantification results of positive stained cells of Ki67 and CD31-positive vesselsin wounds in different groups at day 7. Representative immunohistochemistry staining images (C) and quantification results (D) of VEGF, and α-SMA at day 10 after different treatments. All data were presented as mean ± SD (*n* = 5). **** for *P* < 0.0001.

Angiogenesis in wound tissue plays an important role in the process of wound healing and determines the speed and quality of wound healing 37. The expression of the cell proliferation marker Ki67 in wound tissue was detected using immunohistochemistry 46. The results of immunofluorescence staining showed that on the 7th day after surgery, the expression of Ki67 in the SerMA/RJ-EVs group and the RJ-EVs group was significantly higher than that in SerMA group and the control group (Figure 7A and 7B), which meant that RJ-EVs could potentially accelerate cell proliferation. The immunofluorescence labeling of angiogenesis marker CD31 also verified that SerMA/RJ-EVs could significantly promote angiogenesis *in vivo*; the control group had only a few new blood vessels. The expression levels of vascular endothelial growth factor VEGF, and α-smooth muscle actin (α-SMA) were detected by immunohistochemical staining on the 10th day after surgery to evaluate the angiogenesis of the repaired skin in each group (Figure 7C and 7D). Immunohistochemical staining of CD34 was performed (Figure S18A) and quantified (Figure S18B) to estimate angiogenesis in the wound at day 10. The results showed that the expression of α-SMA in the SerMA/RJ-EVs group was significantly higher than in other groups, which was consistent with the staining results of CD31 and VEGF 47. These results suggested that SerMA/RJ-EVs may promote the formation, regeneration and maturation of new blood vessels by up-regulating the expression of VEGF in wound tissue during wound repair.

Following animal experiments, we conducted tests to verify the *in vivo* biosafety of SerMA/RJ-EVs hydrogels 48. There were no significant differences in whole blood test results between the SerMA/RJ-EVs group and the control group (Figure S19A) and organ tissue section images further confirmed these results. There were no differences in the heart, liver, spleen, lung, and kidney tissue sections between the control group and the SerMA/RJ-EVs hydrogel group (Figure S19B), indicating that the SerMA/RJ-EVs hydrogel group had good biocompatibility *in vivo*.

### Mechanism of SerMA/RJ-EVs in Boosting Wound Healing

**Figure 8:**
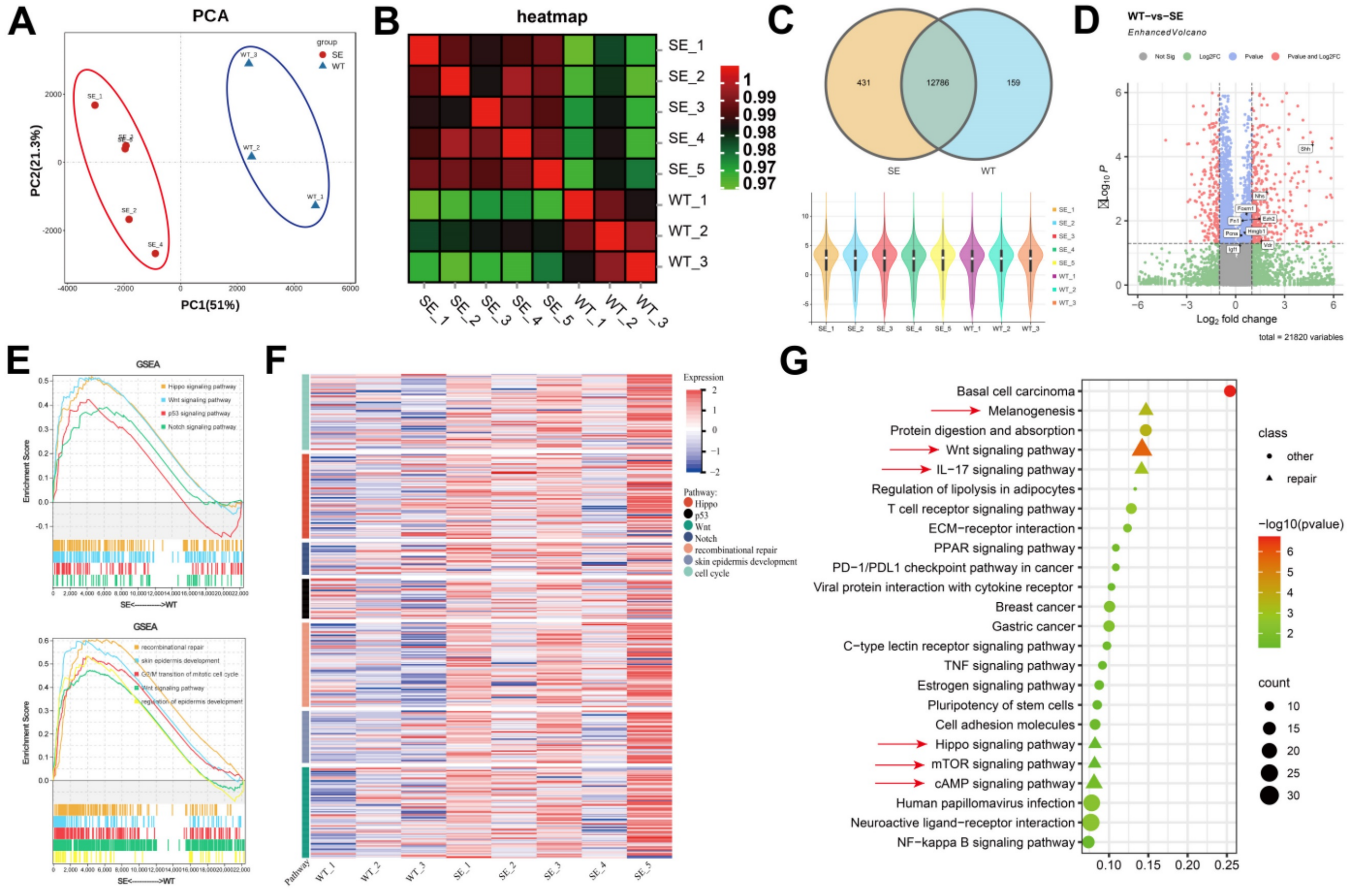
The analysis of skin tissue sequencing. (A) The PCA analysis of the tissue sequencing. (B) The heatmap of correction between SE and WT. (C) The venn and violin diagram of gene counts in SE and WT. (D) The volcano of differentially expressed genes compared SE to WT, the red dots are |fold change| > 2 and adjust *P* value < 0.05, The genes associated with inflammation are marked. (E) The GSEA of the differentially expressed genes in KEGG and GO pathways. (F) The heatmap of gene expression in inflammatory related pathway. (G) The KEGG pathways of the differentially expressed genes, pathways involved in wound healing are marked with red arrow, *P* value < 0.05.

In order to clarify the biological mechanism and dynamic changes of SerMA/RJ-EVs hydrogel-induced rapid wound healing and repair, we performed RNA-seq analysis on samples from the dorsal skin of mice on the 7th day after treatment 49. Based on the gene expression information, we performed Principal Component Analysis (PCA) on the samples. As shown in Figure 8A, the control group (WT) and SerMA/RJ-EVs group (SE) had consistent intra-group and inter-group clustering. Heterogeneity was evident, and the heatmap of relationships between samples also demonstrated similar results (Figure 8B). We performed quality control on each sample, the sequencing depth of each sample was consistent, and the genes sequenced between different groups were highly overlapping (Figure 8C). Through the analysis of differential genes between different groups, we found that proinflammatory factors were generally up-regulated in the SE group. (Figure 8D) 50. To further explore which pathways were involved, we performed the Gene Set Enrichment Analysis (GSEA) on the differential genes (Figure 8E) 51. The two pathways analysis results consistently indicated that the genes in the SE group were involved in inflammatory damage repair-related pathways such as recombinational repair, skin epidemis development, Wnt signaling and Hippo signaling 52. The Hippo pathway is an evolutionarily and functionally conserved signaling pathway that controls organ size by regulating cell proliferation, apoptosis, and differentiation 53. Emerging evidence suggests that the Hippo signaling pathway plays a key role in organ development, epithelial homeostasis, homeostasis, tissue regeneration, wound healing, and immune regulation 54. The gene expression module heatmap also showed that the genes in the SE group were significantly overexpressed in inflammatory injury repair pathways, especially the recombinational repair pathway (Figure 8F). In order to comprehensively analyze the differential pathways between the groups, Kyoto Encyclopedia of Genes and Genomes (KEGG) analysis were performed on the differential genes. We found that there were three repair-related pathways among the top five pathways, including the Wnt signaling pathway (Figure 8G), which was consistent with our previous analysis. These sequencing results elucidated that the Wnt signaling pathway is involved in the skin repair mediated by the SerMA/RJ-EVs hydrogel.

## Conclusions

In summary, we proposed a SerMA/RJ-EVs hydrogel dressing for accelerated wound healing. *In vitro* experiments showed that RJ-EVs had an antioxidant effect, reduced the expression of inflammatory cytokines, and could effectively promote the proliferation and migration of L929 cells. The photo-crosslinked SerMA/RJ-EVs hydrogel dressing could, through sustained release of RJ-EVs, accelerate the process of skin regeneration and wound healing by promoting cell proliferation and angiogenesis. Additionally, we used RNA sequencing of skin tissue to examine the mechanism by which the SerMA/RJ-EVs hydrogel dressing accelerates wound healing. This analysis provided a theoretical framework for its clinical application. Overall, the SerMA/RJ-EVs hydrogel dressings have great clinical potential for accelerating wound healing and may be a good substitute for current wound dressings.

## Materials and Methods

### Materials

Methacrylic anhydride was purchased from Sigma-Aldrich (USA). Lithium phenyl-2,4,6-trimethylbenzoylphosphinate (LAP) was obtained from Aladdin (Shanghai, China). Silkworm cocoons (Bombyx mori) were purchased from Suzhou Siruibao Biotechnology Co., Ltd. Royal jelly was purchased from Hangzhou Beewords Apiculture Co., Ltd. Other conventional chemical reagents were purchased from Sinopharm Group Chemical Reagent Co., Ltd (China).

### Cell Lines and Animals

Mouse fibroblasts L929 cells were obtained from the Cell Bank of the Chinese Academy of Sciences (Beijing, China), and were cultured in DMEM containing 10% fetal bovine serum and 1% penicillin streptomycin at 37°C in a humidified incubator with 5% carbon dioxide. Eight-week-old female mice (BALB/c) were purchased from Zhuhai Baishitong Biotechnology Co., Ltd. All animal experiments and animal care were conducted under protocols approved by the Regional Ethics Committee for Animal Experiments at Shenzhen Bay Laboratory (Permit No. AERL202201).

### Isolation of RJ-EVs

Fresh royal jelly was homogenized in a grinder with PBS for 30 minutes, and then stored in a refrigerator at 4°C overnight, so that the active ingredients in royal jelly could be more fully dissolved and released into PBS. To remove large particles of royal jelly, the royal jelly PBS suspensions were obtained by differential centrifugation after 10, 20, and 30 min at 500 g, 2,000 g, and 10,000 g, respectively. After centrifugation, the pellet was discarded, and the supernatant was filtered through a 0.22 μm filter 55. Subsequently, the filtrate was centrifuged at 110,000 g for 120 min. After centrifugation, the supernatant was discarded, and the pellet was resuspended by sonication and dispersed in PBS. For purification of RJ-EVs, the resuspension was centrifuged at 110,000 g for 120 min 56. After centrifugation, the pellet was resuspended by sonication and dispersed in 300 μL PBS. The protein concentration of the obtained RJ-EVs were determined with a BCA protein assay kit and stored at -80°C for further use.

### Characterization of RJ-EVs

The morphology of RJ-EVs was examined by TEM (JEOL, JEM-2100F). Malvern Zetasizer Nano ZS90 (Malvern, UK) was used to measure DLS. The particle size and concentration of RJ-EVs were measured using NTA with ZetaView PMX 110 (Particle Metrix, Meerbusch, Germany). The total protein of RJ-EVs was detected by SDS-PAGE.

### Cellular Uptake

RJ-EVs were labeled with the lipophilic dye DiO green (Beyotime Biotechnology, China). Briefly, the L929 cells were cultured with 25 μg/mL DiO-labeled RJ-EVs and fixed with 4% paraformaldehyde for 15 min at room temperature. The L929 cells were then washed with PBS, and the cell nuclei were stained with DAPI at room temperature for 15 min. Finally, the L929 cells were observed with confocal microscopy (Nikon A1, Japan). In addition, the cellular uptake of RJ-EVs at different concentrations (*e.g.*, 0, 10, 25, and 50 μg/mL) was investigated by flow cytometry (Cyto FLEX; Beckman Coulter).

### CCK-8 Assay

The cell viability of the L929 cells was assessed with a Cell Counting Kit-8 (CCK-8, Dojindo, Japan) assay. The specific experimental methods are reported in a previous article 39. Briefly, the L929 cells were seeded into 96-well plates and given different treatments. Then 100 μL/well of CCK-8 solution was added to 96-well plates. The cells were then incubated for a period of time. The absorbance at 450 nm was detected with a microplate reader (BioTek, USA).

### Scratch Test

A cell scratch test was used to detect the effect of different concentrations of RJ-EVs (10, 25, 50μg/mL) on the migration ability of L929 cells. After reaching 90% confluence, the monolayer was scratched with a 200 μL pipette tip. The cells were washed with PBS and exposed to different treatments. Then, cells were observed under microscope (CKX53, Olympus, Tokyo, Japan) at 0, 6, 12, and 24 h. The percentage of wound closure was calculated using the ImageJ software.

### *In vitro* Antioxidant Property Evaluation

The PTIO radical scavenging rate of the RJ-EVs was determined 57. A freshly prepared PTIO aqueous solution (10 mg/mL) was added to a 96-well plate (100 μL per well), followed by the addition of RJ-EVs at different concentrations (*e.g.*, 10, 15, 20, 25, 50, and 100 μg/mL). Subsequently, the PTIO solution was incubated at 37°C in the dark for 30 min, and its absorbance at 557nm was measured using a microplate reader. The formula used for calculating the clearance rate was:



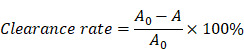



(Where A_0_ represents a sample volume of 0 μL; A represents a sample volume of *x* μL).

### Enzyme-Linked Immunosorbent Assay (ELISA)

The effects of RJ-EVs on the expressions of pro-inflammatory factors IL-6, TNF-α and anti-inflammatory factors IL-10 and TGF-β1 were detected by an ELISA kit. Mouse-derived macrophages (RAW 264.7) were stimulated with lipopolysaccharides and then incubated with RJ-EVs at different concentrations (10, 25, and 50 μg/mL) for 24 h. Then, the supernatant was collected for further use. The specific operation steps were carried out in strict accordance with the instructions of the ELISA kit.

### Preparation of SerMA

A heat and alkaline degumming method was used to extract sericin from natural silkworm cocoons. Ultra-pure water was used to clean natural cocoons. The washed cocoons (10 grams) were boiled in 0.02 M Na_2_CO_3_ solution (400 mL) for 1 h, and then dialyzed against ddH_2_O with a dialysis bag with relative molecular weight of 3.5 kDa to remove Na_2_CO_3_, and finally sericin was obtained by freeze-drying. The method of using MA to modify sericin is consistent with previous reports 58.

### Preparation and Characterization of SerMA/RJ-EVs

The precursor hydrogel solutions were prepared by mixing RJ-EVs (25 μg/mL), LAP (5 mg/mL), and SerMA (5 mg/mL) in PBS. The solution was then irradiated with a 405 nm laser for 40 s to obtain the SerMA/RJ-EVs composite hydrogel dressing. The porous morphology of the hydrogel was observed by scanning electron microscopy (SEM, JSM 6100, Jeol, Japan) Rheology was analyzed with an MCR-92 rheometer (Anton Paar, Austria). The universal mechanical test machine was produced by the Changchun New Testing Machine Co., Ltd.

### Sustained Release of RJ-EVs from SerMA/RJ-EVs

Firstly, *in vitro* RJ-EVs release study was performed. The precursor hydrogel solutions containing RJ-EVs (25 μg/mL) and SerMA (5 mg/mL) were prepared. The SerMA/RJ-EVs hydrogels were prepared using 100 μL of precursor hydrogel solution, and subsequently incubated with PBS at 37°C. The supernatant was aspirated at different time points and analyzed using a fluorescence spectrometer, with fluorescence excitation at 754 nm and emission at 778 nm. Then, *in vivo* RJ-EVs release studies were performed. A wound model with a diameter of 8 mm was made on the skin of BALB/c mice with sterilized scissors. The mice were divided into 2 groups, with 3 mice in each group carrying SerMA, and SerMA/RJ-EVs. The SerMA hydrogel and SerMA/RJ-EVs composite hydrogel were formed by dropping 100 μL of the precursor hydrogel solution onto the skin wound and then irradiated with a 405 nm laser for 40 s. The RJ-EVs were imaged by a small animal living imaging system at different time periods.

### *In vivo* Healing of Full-Thickness Wound by Different Gels

For the purpose of creating mouse models for full-thickness skin damage, twenty 6-week-old female BALB/c mice were randomly divided into four groups: the control group, RJ-EVs group, SerMA group, and SerMA/RJ-EVs group. The mice were anesthetized and fixed on the operating table after hair removal with an animal anesthesia machine. After the skin was sterilized with alcohol, a round cortical excision wound with a diameter of 8 mm was created on the dorsal skin with sterile surgical instruments. After the wound model was successfully established, different treatments were adopted for different groups. The back wounds of all mice were recorded with cameras at day 0, 4, 7, 10 and 14. The wound area and wound healing rate of each group of mice were analyzed by Image J based on methods in a previous report 41. The wound tissue specimens were collected at day 14 post-treatment and fixed with 4% paraformaldehyde. Fixed specimens were embedded in paraffin and sectioned into slices. The obtained sections were stained with H&E, Masson, CD34, VEGF, and α-SMA. Furthermore, immunohistochemical (IHC) analysis was performed by staining the sections with the markers Ki67 and CD31 at day 7 and observing these sections with a light microscope.

### Skin Tissue Sequencing

On the 7th day after the establishment of the mouse full-thickness skin injury model, the wounds of the mice in the SerMA/RJ-EVs group and the control group were excised and placed in cryopreservation tubes, and frozen in liquid nitrogen for 5 minutes. Sequencing analysis was performed by Guangzhou Kedio Biotechnology Co., Ltd. (Guangzhou, China). These procedures were performed as previously described. All sequencing results were analyzed based on R version 4.2.0 (version 4.2.0, https://www.r-project.org/), where gene expression (Counts) differential analysis was performed using the R package "edgeR", |fold Change|> 2 and adjusted *P* < 0.05 were considered significant differences in gene expression 59, 60. The KEGG pathway analysis was performed with the R package "clusterprofiler", the heatmap was drawn with the "pheatmap" R package, and the rest of the graphs were drawn with the "ggplot2" package and GraphPad Prism 9.

### Statistical Analysis

Statistical analysis was performed using GraphPad Prism software (version 9). Statistical differences were presented according to the *P* values and represented as follows: * for *P* < 0.05, ** for *P* < 0.01, *** for *P* < 0.001, and **** for *P* < 0.0001.

## Supplementary Material

Supplementary figures.
