# Supplementary material for: In situ formed scaffold with royal jelly-derived extracellular vesicles for wound healing
Source: Theranostics. 2023 May 8;13(9):2811–24. doi: 10.7150/thno.84665 (PMC10240823; doi:10.7150/thno.84665)
Supplement: Supplementary file 1 — Supplementary figures. [file thnov13p2811s1.pdf]

Supporting Information

In situ formed scaffold with royal jelly-derived extracellular vesicles for wound healing

Dehong Tan[1,2,3], Wenxiang Zhu[2,4], Lujie Liu[2], Yuanwei Pan[2], Yangtao Xu[2,5], Qinqin Huang[1], Lingling Li[3], and Lang Rao[1,2,*]

[1] The Second Affiliated Hospital of Zhengzhou University, Academy of Medical Sciences, Zhengzhou University, Zhengzhou 450052, China.

[2] Institute of Biomedical Health Technology and Engineering, Shenzhen Bay Laboratory, Shenzhen 518132, China.

[3] Department of Pharmaceutics, School of Pharmacy, Nanjing Medical University, Nanjing 211166, China.

[4] College of Materials Science and Engineering, Hunan University, Changsha 410082, China.

[5] Cancer Center and Department of Oncology, Renmin Hospital of Wuhan University, Wuhan 430060, China.

* Corresponding author: lrao@szbl.ac.cn (L.R.)

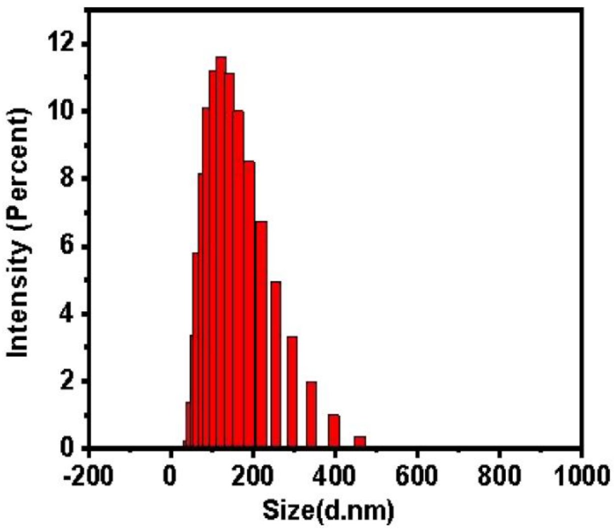

Figure S1. The size distributions of purified RJ-EVs were measured using DLS and the diameters were 100 ± 0.78 nm.

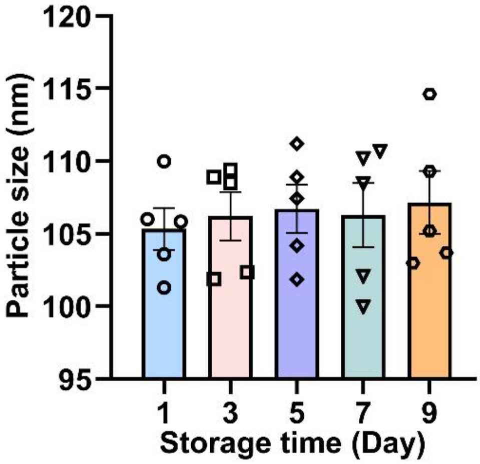

Figure S2. Hydrodynamic size change diagram of RJ-EVs in PBS for 9 days.

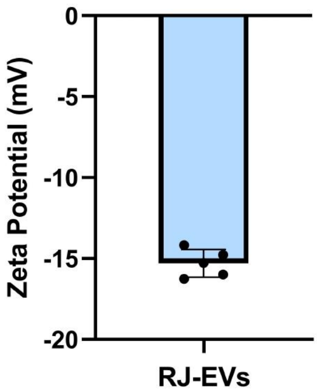

Figure S3. The zeta potential of purified RJ-EVs measured by the DLS.

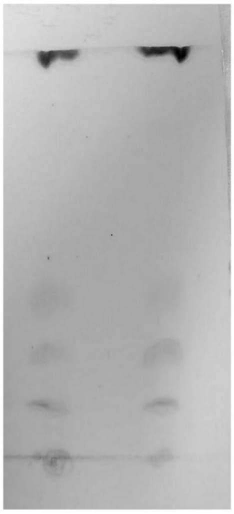

Figure S4. The TLC analysis of RJ-EVs lipids.

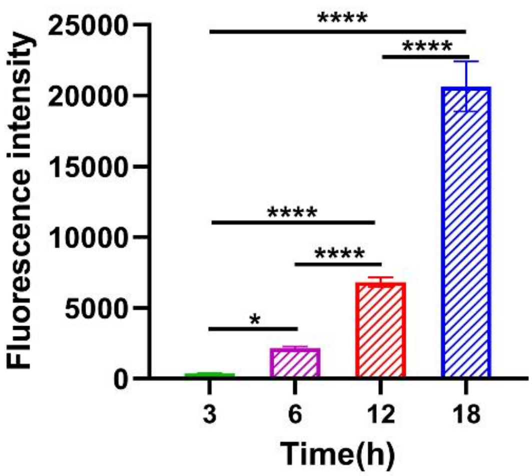

Figure S5. Quantitative analysis of cellular uptake.

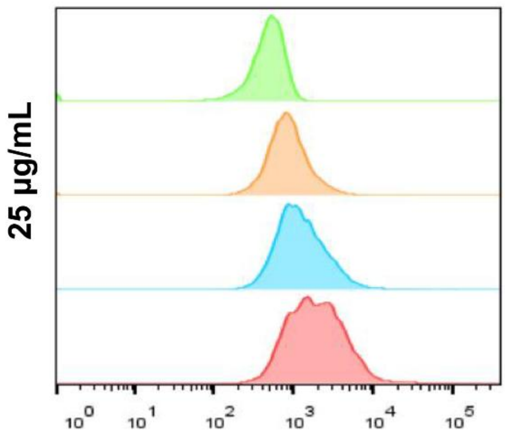

Figure S6. Flow cytometry detection of RJ-EVs at a concentration of 25 μg/mL.

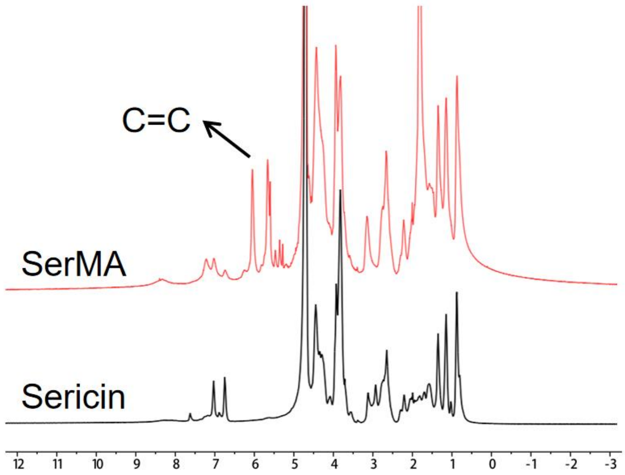

Figure S7. The 1H NMR spectra of Sericin and SerMA in D_2O.

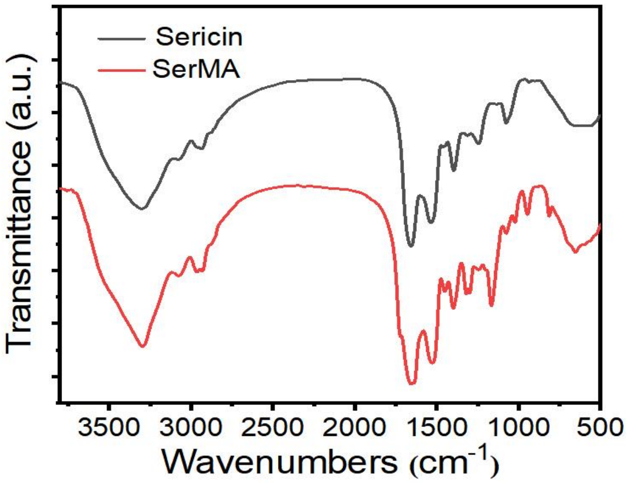

Figure S8. The FTIR spectra of Sericin and SerMA.

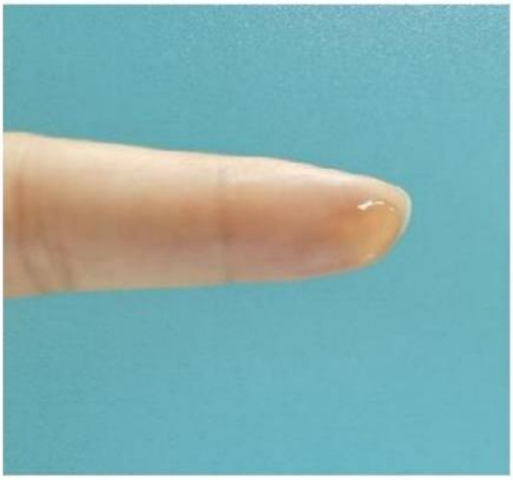

Figure S9. The SerMA/RJ-EVs hydrogel can be firmly attached to the skin surface.

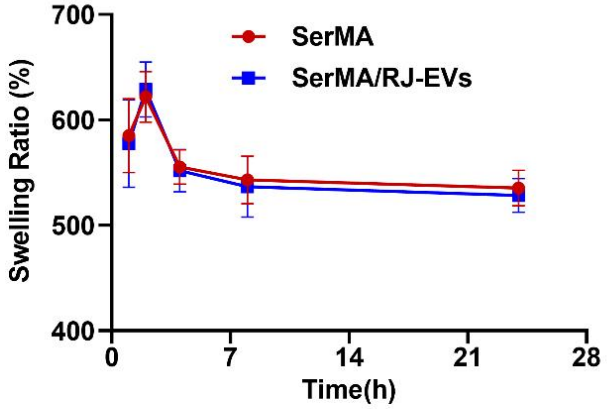

Figure S10. The swelling rates of SerMA and SerMA/RJ-EVs.

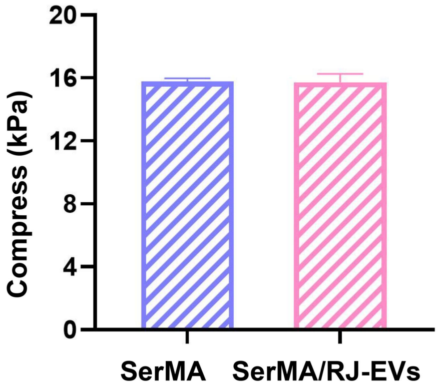

Figure S11. The mechanical properties of SerMA and SerMA/RJ-EVs.

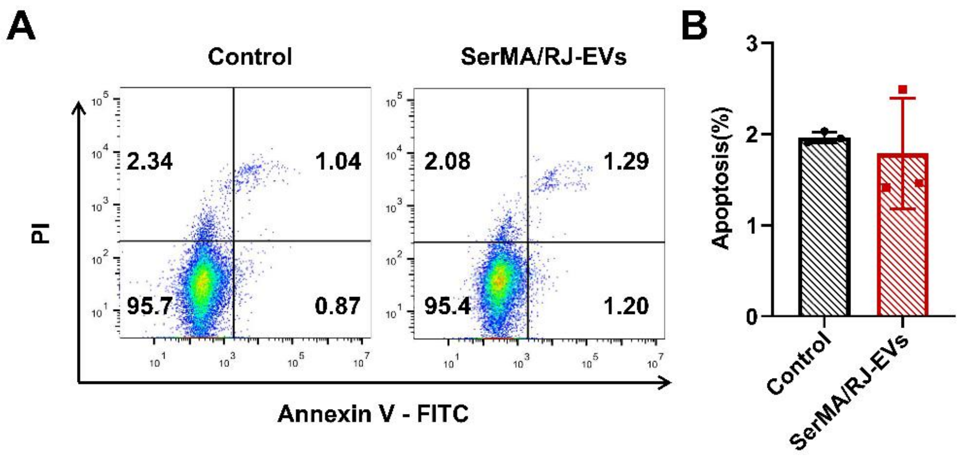

Figure S12. Representative images (A) and quantification results (B) of the Calcein-AM/PI staining of L929 cells after SerMA/RJ-EVs treatment.

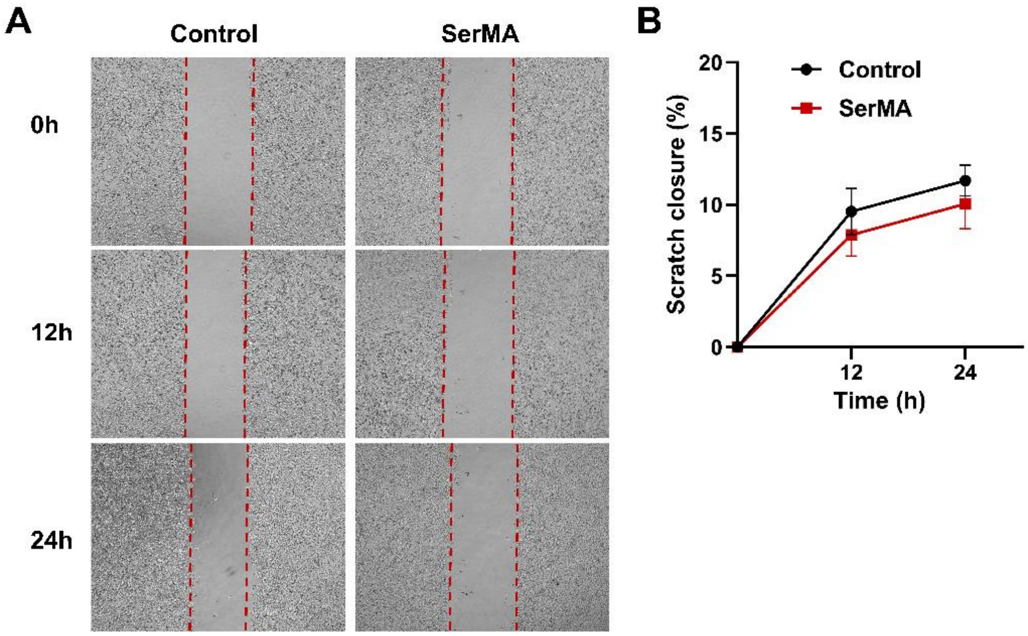

Figure S13. Cell migration-promoting ability of SerMA. (A) Cell migration diagram of the control group and the SerMA group. (B) The scratch closure rate of the control group and the SerMA group.

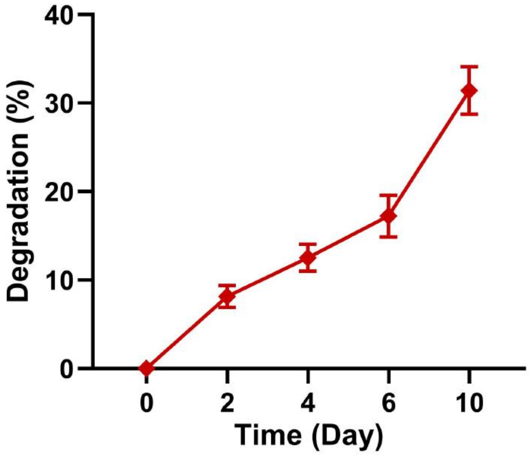

Figure S14. *In vitro* degradation profiles of SerMA/RJ-EVs hydrogel.

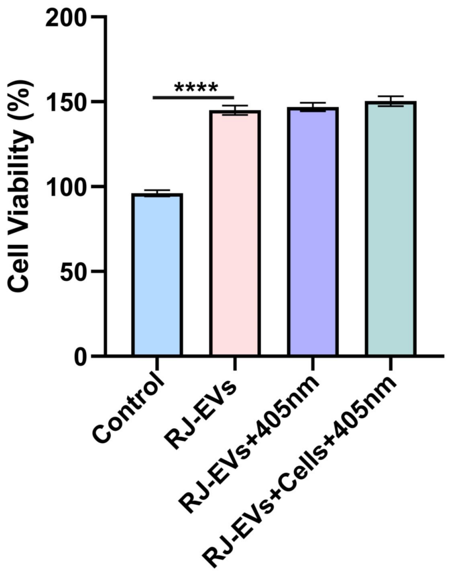

Figure S15. The cytotoxicity of RJ-EVs exposed to 405 nm blue light.

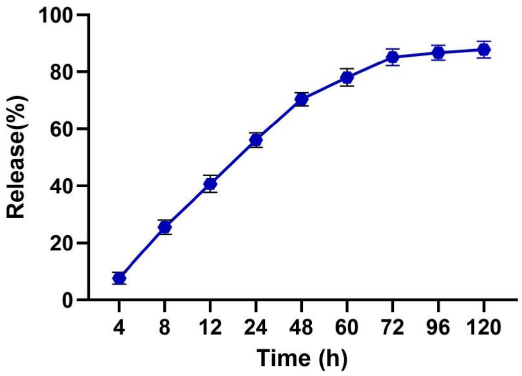

Figure S16. The release profile of SerMA/RJ-EVs hydrogel.

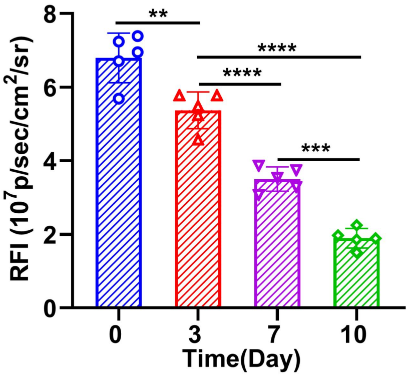

Figure S17. The relative fluorescence intensity of the SerMA/RJ-EVs group at different time.

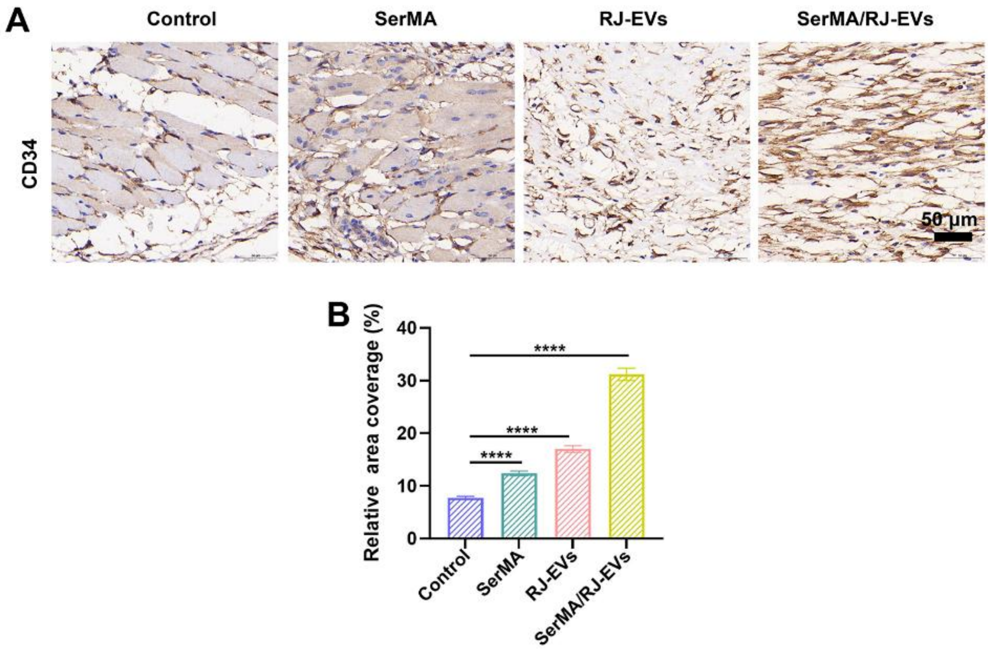

Figure S18. Representative immunohistochemistry staining images (A) and quantification results (B) of CD34 in the wound bed on day 10 after different treatment.

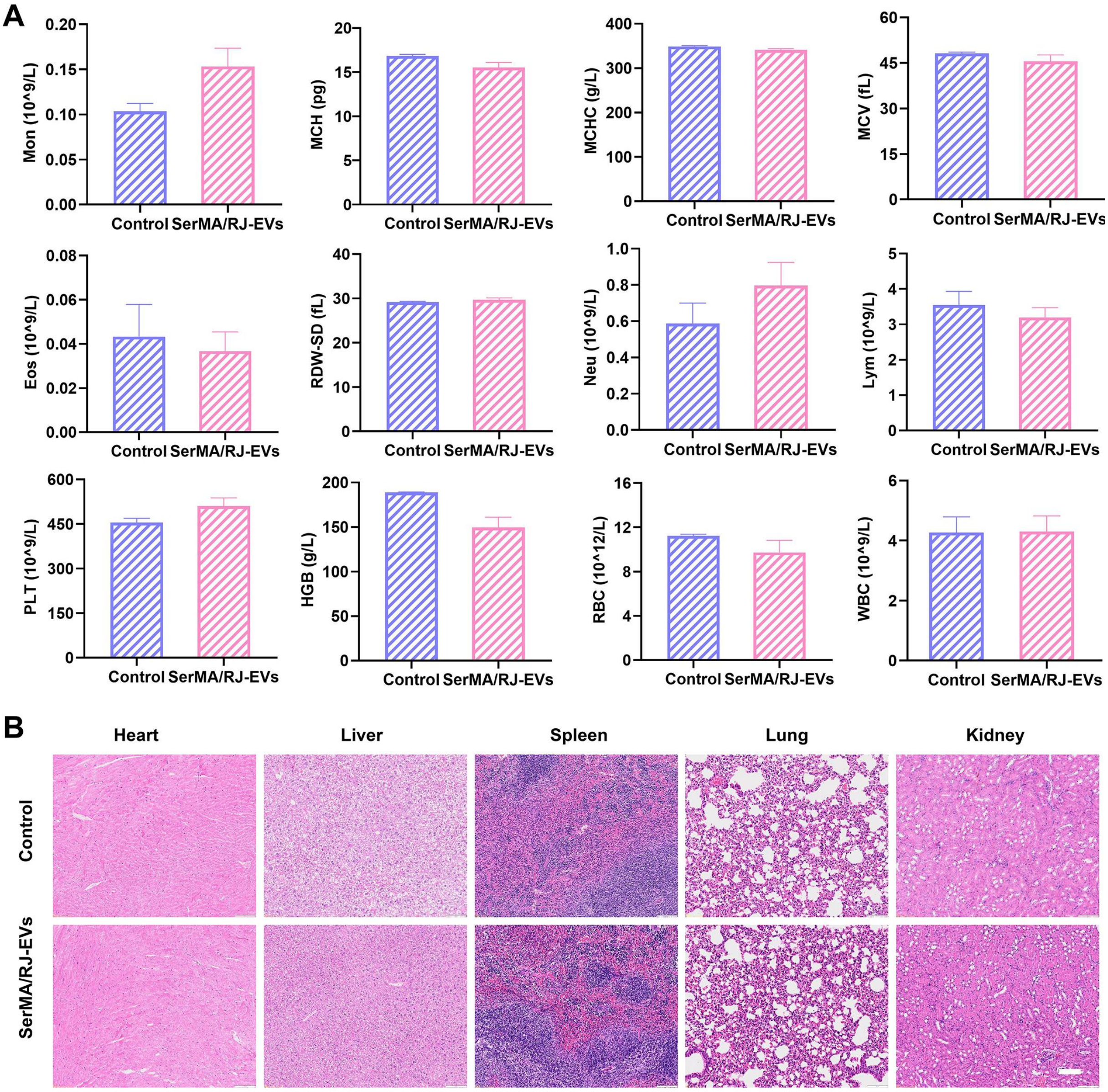

Figure S19. Safety evaluation of SerMA/RJ-EVs. (A) Routine blood tests and blood biochemistry of mice after various therapies. (B) H&E staining of the main organs (*i.e.,* heart, liver, spleen, lung, and kidney) of mice after different treatment.
